# Evaluation of intracellular telomerase activity through cascade DNA logic gates†
†Electronic supplementary information (ESI) available: Sequences used in this study, fluorescence spectroscopy of logic gate activation using synthetic TS oligonucleotide with different numbers of elongation repeats, flow cytometry data, confocal images of counter staining, time course, control samples, and L-02 and Hep G-2 cells. See DOI: 10.1039/c6sc01953f



**DOI:** 10.1039/c6sc01953f

**Published:** 2016-08-01

**Authors:** Wenjing Wang, Shan Huang, Jingjing Li, Kai Rui, Sai Bi, Jian-Rong Zhang, Jun-Jie Zhu

**Affiliations:** a State Key Laboratory of Analytical Chemistry for Life Science and Collaborative Innovation Center of Chemistry for Life Sciences , School of Chemistry and Chemical Engineering , Nanjing University , Nanjing 210093 , China . Email: jrzhang@nju.edu.cn ; Email: jjzhu@nju.edu.cn; b School of Chemistry and Life Science , Nanjing University Jinling College , Nanjing 210093 , China; c School of Medical Imaging , Xuzhou Medical University , Xuzhou 221006 , China; d School of Pharmacy , Ningxia Medical University , Yinchuan 750004 , China; e College of Chemistry and Chemical Engineering , Collaborative Innovation Center for Marine Biomass Fiber Materials and Textiles , Qindao University , Qingdao 266071 , China

## Abstract

Telomerase plays a vital role in cancer and aging, and telomerase activity detection has drawn great attention recently. However, a feasible *in situ* imaging system for intracellular telomerase is still a challenge. Here, we develop a novel approach to image intracellular telomerase activity using DNA-based computation. A cascade nucleic acid logic gate that responded to intracellular telomerase was constructed. A telomerase substrate (TS) probe, extended by intracellular telomerase, worked as an input to initiate computation cascades. In this way, intracellular telomerase could be clearly indicated by fluorophore labeled nucleic acids as the output. Through one-step incubation, evaluation of the intracellular telomerase activity for a HeLa cell line and the ability to differentiate cancer cells from normal cells could be realized. Furthermore, the response of intracellular telomerase activity to a telomerase-inhibiting model drug was observed using the proposed method. Thus, this intracellular telomerase computation device will allow improvements in studying the relationship between telomerase and cancer, and may help to develop telomerase inhibitors. This finding also expands the applications of DNA computational techniques in cells.

## Abstract 2

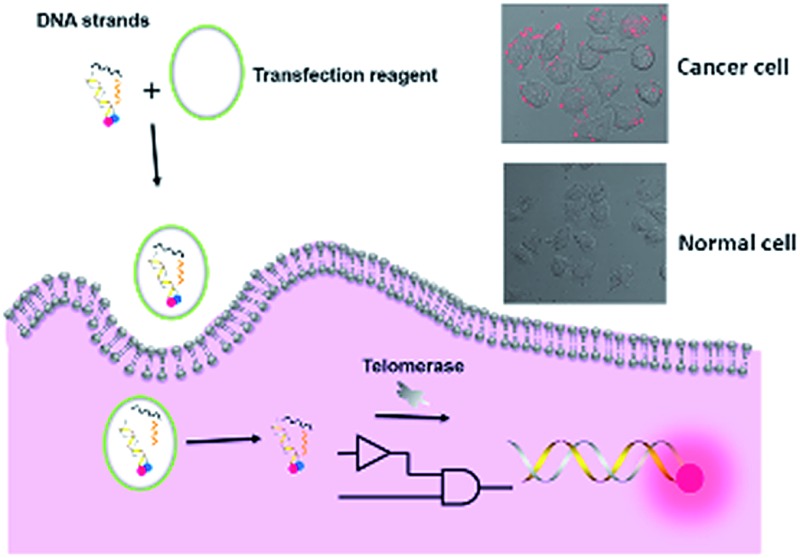
DNA computation allowed the imaging of intracellular telomerase and enabled the differentiation of cancer cell lines and normal cell lines.

## Introduction

Telomerase is a ribonucleoprotein which can maintain the length of a chromosome by adding repetitive nucleotide sequences (TTAGGG for vertebrates) to the 3′ end of the chromosome, leading to the endless division of cancer cells.^1–5^ Telomerase plays a vital role in human cancer, and it has been reported that telomerase is overexpressed in more than 85% of cancer cells. It has been widely recognized as an important biomarker for cancer and a potential therapeutic target.^6–8^

Currently, polymerase chain reaction (PCR)-based telomeric repeat amplification protocol (TRAP) and its modified assays are the most popular methods to evaluate telomerase activity in cell extracts and tissues.^6,9,10^ Although they have excellent sensitivity, the relatively complex detection process and the intrinsic drawbacks of PCR-based assay, including the risk of carry-over contamination and susceptibility to polymerase inhibition by cell extracts, have led to the development of many alternative PCR-free methods, including colorimetric,^11–13^ fluorescence,^14–16^ electrochemical^17–19^ and electroluminescence^20–22^ methods. While these approaches have allowed the evaluation of telomerase activity even in clinical use, they are thus far limited to cell extracts. In order to observe the response of telomerase activity to inhibitors or other drugs immediately or to obtain information on telomerase activity at the single cell level, *in situ* detection methods based on gold nanoparticles (AuNPs) and mesoporous silica nanoparticles have been proposed.^23–25^ Although satisfactory results have been achieved, the complicated preparation process of oligonucleotide modified AuNPs and the non-specific release of mesoporous silica nanoparticles have hampered their further use in clinical diagnosis. Thus, constructing a feasible *in situ* imaging system for intracellular telomerase is still a challenge.

DNA computation uses nucleic acid strands as inputs and outputs to operate DNA-based digital logic circuits, perform complex information processing and fulfil sophisticated control tasks. Since the first DNA-based computer appeared in 1994,^26^ this area has attracted considerable interest from researchers all over the world. Until now, DNA-based computers have been designed to respond to different oligonucleotide inputs for a variety of biochemical applications, such as the identification of disease-related mRNA and control of gene expression,^27^ operation of RNAi-based evaluators in cells with gene expression outputs,^28^ pH sensing in a living organism,^29^ identification of specific cancer cells,^30^ and cancer recognition and therapy.^31^ The basic principle of DNA computation relies exclusively on the sequence recognition of Watson–Crick base pairing and strand displacement. Recently, specific microRNA (miRNA) in live mammalian cells has been used as an input to operate a designed AND logic gate to image intracellular miRNA and monitor changes in miRNA profile responding to expression regulators.^32^

Here, we demonstrate that beyond miRNA, intracellular telomerase can be used as an input to operate the cascade logic gate *via* DNA computation. The output of the cascade logic gate is a fluorophore-labelled strand, allowing the system to reflect telomerase activity without cell lysis. This method can work as a useful tool to image telomerase in cancer cells as well as to monitor the response of telomerase to telomerase-inhibiting model drugs in real-time. Although molecular beacons have the potential to be rationally designed to finish this task, DNA computation in live cells allows for logic operation with DNA strand inputs, and the generated oligonucleotide outputs could be incorporated with other applications for the next step.

## Results and discussion

### Principle of cascade DNA logic gate operation

**Scheme 1:**
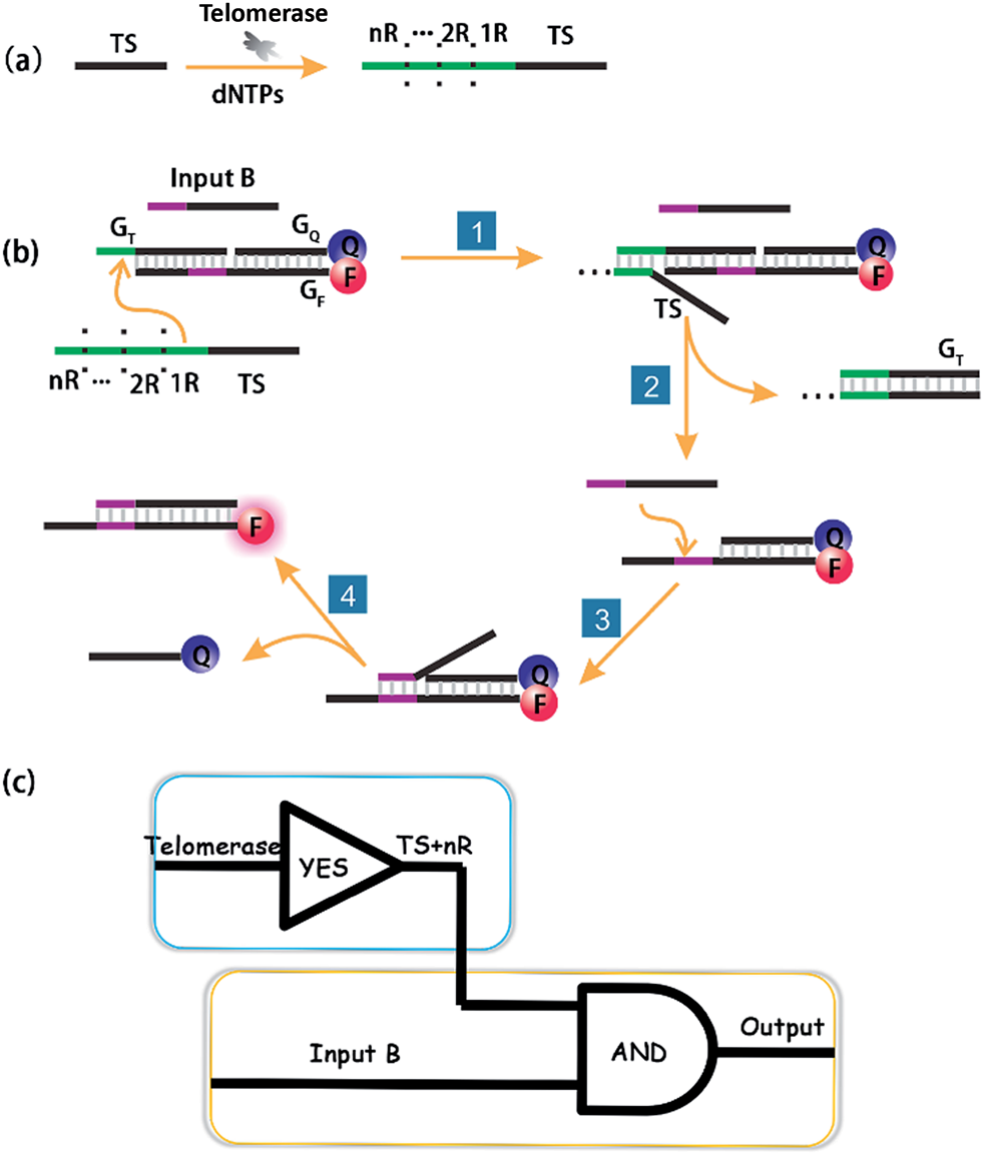
Schematic illustration of DNA computation for imaging intracellular telomerase. (a) Upon extension by telomerase in cells, (b) the extended TS probe (TS + *n*R) and Input B strand worked as inputs to initiate toehold-mediated strand displacement, separating a fluorophore and quencher modified DNA duplex, and producing a fluorescence signal. (c) A cascade logic circuitry that is composed of one YES gate and one AND gate enables the imaging of intracellular telomerase with one single step incubation.

According to the sequence of the telomerase elongation product, the telomerase-based logic gate was rationally engineered. The principle of the method is illustrated in Scheme 1. The whole system of telomerase-based DNA computation includes the TS + *n*R strand, which is the telomerase substrate (TS) primer extended by intracellular telomerase, a toehold-bearing DNA duplex that consists of a G_T_ strand, a fluorophore-modified G_F_ strand and a quencher-modified G_Q_ strand, and Input B which is the partial-complementary strand of G_F_. Initially, the TS probe, Input B strand and toehold-bearing DNA duplex were transfected into cells at the same time, and could coexist stably in the absence of telomerase. When the TS probe was extended by telomerase in cells with repetitive sequences of TTAGGG, it could recognize and hybridize with the toehold domain of the duplex to initiate spontaneous strand migration and displacement, thereby separating the fluorophore and the quencher, producing a fluorescence signal indicating the presence of telomerase. However, in a cell line without expressed telomerase activity it should fail to yield such a fluorescence signal because of the lack of extension of the TS probe which is necessary for the toehold displacement. As the same telomere sequence is included within the 3′ end of G_F_, the 3′ end of the G_F_ strand was modified with a phosphate group to avoid extension of the G_F_ strand by telomerase.

Importantly, the proposed strategy can be considered as a cascade logic circuit that is composed of one YES gate and one AND gate (Scheme 1c). Specifically, intracellular telomerase works as the specific input to activate the first YES gate. The output of this YES gate (TS + *n*R) and Input B strand together perform the following AND gate computation. This cascade DNA logic operation enables the imaging of intracellular telomerase with one single step incubation.

### Feasibility test *in vitro*

**Fig. 1:**
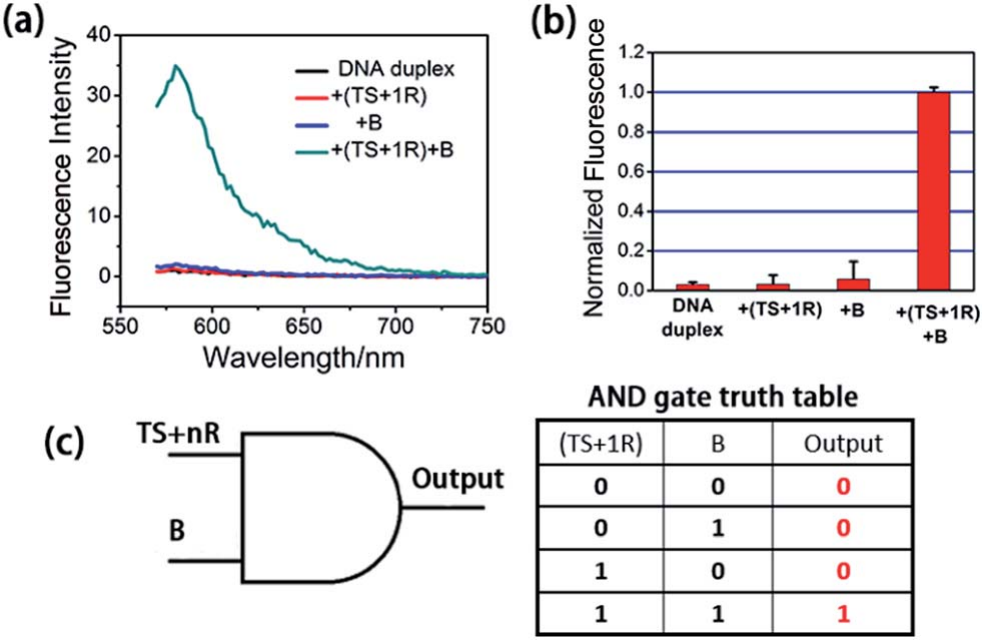
Activation test of the AND logic gate with synthetic telomerase elongation products TS + 1R and Input B strands *in vitro*. (a) Fluorescence spectra recorded using a microplate reader under different situations. The excitation wavelength was 550 nm. The DNA duplex was the toehold-bearing duplex. (b) Histograms of the maximum emission wavelength at 580 nm. The fluorescence signal of TAMRA was measured after 3 h incubation and normalized. Error bars represent the standard deviation from three independent measurements. Both of the two inputs were required for the activation of the AND gate. (c) Graphic symbol and truth table for the two inputs of the AND gate.

At first, we employed the synthetic telomerase elongation product TS + 1R, which is the TS probe extended by one telomere repeat, to test the feasibility of the proposed method *in vitro*. The toehold-bearing DNA duplex was initially purified using polyacrylamide gel electrophoresis and then incubated with the synthetic telomerase elongation product and Input B strand. As shown in Fig. 1a and b, both of the two inputs were required to activate the AND gate for telomerase to produce a fluorescence signal, whereas only a single input could not operate the logic gate. Fig. 1c is a truth table of the AND gate, showing that the telomerase elongation product (TS + *n*R) and Input B strand were essential to initiate the AND gate operation to give an output signal. In order to assess whether multiply-elongated sequences could be recognized with the same system, the synthetic telomerase elongation products TS + 2R and TS + 3R, corresponding to the TS probe extended by two and three telomere repeats were also introduced. As shown in Fig. S1,† fluorescence recovery for both TS + 2R and TS + 3R strands was observed, with the same intensity as in the singly-extended oligonucleotide, suggesting that this method works equally well for TS oligonucleotides with one or more TTAGGG repeats, validating the assay for specific detection of telomerase activity. Notably, compared with the most widely used TRAP assay and the existing *in situ* imaging methods, our proposed approach could realize the detection of the short telomerase elongation product TS + 1R. TRAP, the most popular and widely used telomerase activity evaluation method for cell lysate cannot fulfill this task, since the downstream primer CX involved in the PCR process needs at least three extended repeats to bind.^6^ The mesoporous silica nanoparticle-based^23^ and gold nanoparticle-based^24^ *in situ* telomerase activity detection methods require at least six and three extended repeats, respectively, to achieve structure switching to give a fluorescence signal.

In order to further demonstrate the feasibility of our proposed method, we incubated the Input B strand and TS probe with telomerase extracts from 5000 cells. The fluorescence recovery was observed clearly while the control experiment using lysis buffer only didn't show increasing fluorescence, validating that the TS probe was extended by telomerase from the cell extracts and that the strand displacement reaction was performed subsequently (Fig. S2†).

### Telomerase activity imaging in cells using DNA computation

**Fig. 2:**
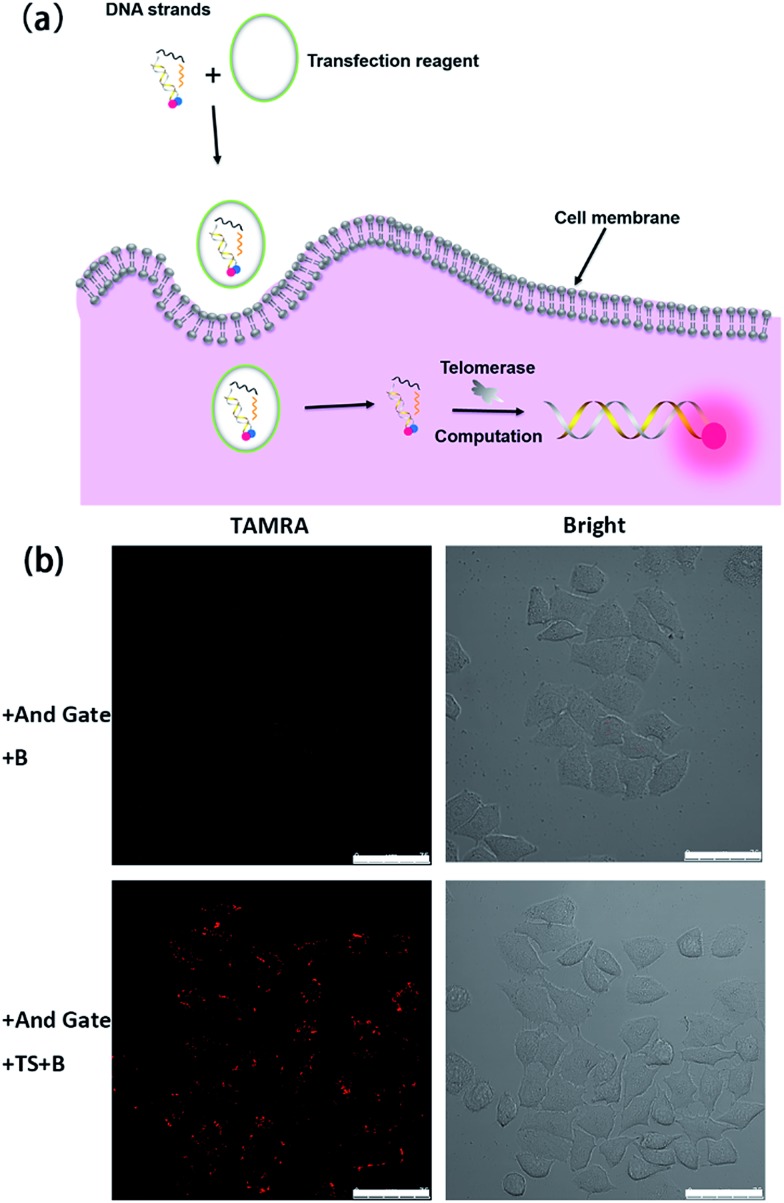
Activation test of the cascade DNA logic gate in HeLa cells. (a) Schematic illustration of the evaluation of intracellular telomerase using the cascade DNA logic gate. (b) Confocal images of HeLa cells for the verification of logic gate activation responding to intracellular telomerase with the toehold-bearing DNA duplex and Input B strand (top) and with the toehold-bearing DNA duplex, TS strand and Input B strand (bottom) transfected for 3 h. Scale bar: 75 μm.

Having demonstrated the successful computation operation of the AND gate *in vitro*, we then studied its imaging capability in cancer cells by using human cervical cancer cells (HeLa cells) as a model. DNA strands were transfected into HeLa cells using the transfection reagent X-tremeGENE (Roche). The control experiment was done with transfection of the Input B strand and the toehold-bearing duplex into the HeLa cells for 3 h. Since only one input is not enough to activate the AND gate, no fluorescence signal was observed (Fig. 2b). However, for the transfection of both inputs (TS probe and Input B strand) together with the toehold-bearing duplex, the TS probe was extended by intracellular telomerase and the strand displacement reaction occurred to show a clear fluorescence signal, showing the feasibility of the method for imaging intracellular telomerase. This also indicated that the TS segment within G_F_ was unchanged, and thus TS elongation together with the Input B strand could activate the AND gate. Flow cytometry was also used to analyze the fluorescence of cells (Fig. S3†). Compared with HeLa cells transfected with only the Input B strand and the toehold-bearing DNA duplex, the doubly-transfected HeLa cells (with both Inputs) showed an enhanced fluorescence signal, which is in good agreement with the confocal microscopy results. Counter staining with DAPI indicated that the activated cascade logic gate fluorescence signal was mainly distributed around the nuclear periphery, suggesting high telomerase activity in the cytoplasm of cancer cells (Fig. S4†) as reported elsewhere.^23^

A time course experiment was performed to investigate the proper amount of incubation time required for logic gate activation by intracellular telomerase in cells (Fig. S5†). After transfection with the TS probe, Input B strand and toehold-bearing DNA duplex for 0.5 h, no detectable fluorescence signal was observed. After 1.5 h transfection, the intensity of the fluorescence increased gradually in the cytoplasm of the cells due to the successful transfection of DNA strands and the subsequent DNA computation-based imaging of intracellular telomerase. A time of 3 h was enough to observe a robust fluorescence signal caused by intracellular telomerase activity. So this incubation time was chosen for the following cellular study.

We then transfected HeLa cells with the toehold-bearing DNA duplex alone, to investigate whether the duplex was degraded by intracellular nucleases. Within the detection time course of 3 h, there was no detectable fluorescence signal in the cells (Fig. S6†), which is in agreement with previous reports.^32^ Thus, this result indicated that the fluorescence signal came from the logic gate activation, and not from the background signal of the duplex degradation. Thus, the cascade DNA logic gates could be used to accurately image intracellular telomerase without interference from cellular nucleases and proteins. To further test the feasibility for intracellular telomerase imaging with DNA computation, we further chose human hepatocyte carcinoma cells (Hep G-2) and negative contrast-human normal liver cells (L-02) as a pair of model cell lines for imaging. After transfection for 3 h, a fluorescence signal in the Hep G-2 cells was observed, while no detectable signal was observed in the L-02 cells (Fig. S7†), consistent with the known expression of telomerase in the cancer cell line, rather than in the normal cell line. Thus, this method can be successfully used to distinguish cancer cells from normal cells.

### Telomerase activity of different cell lines

**Fig. 3:**
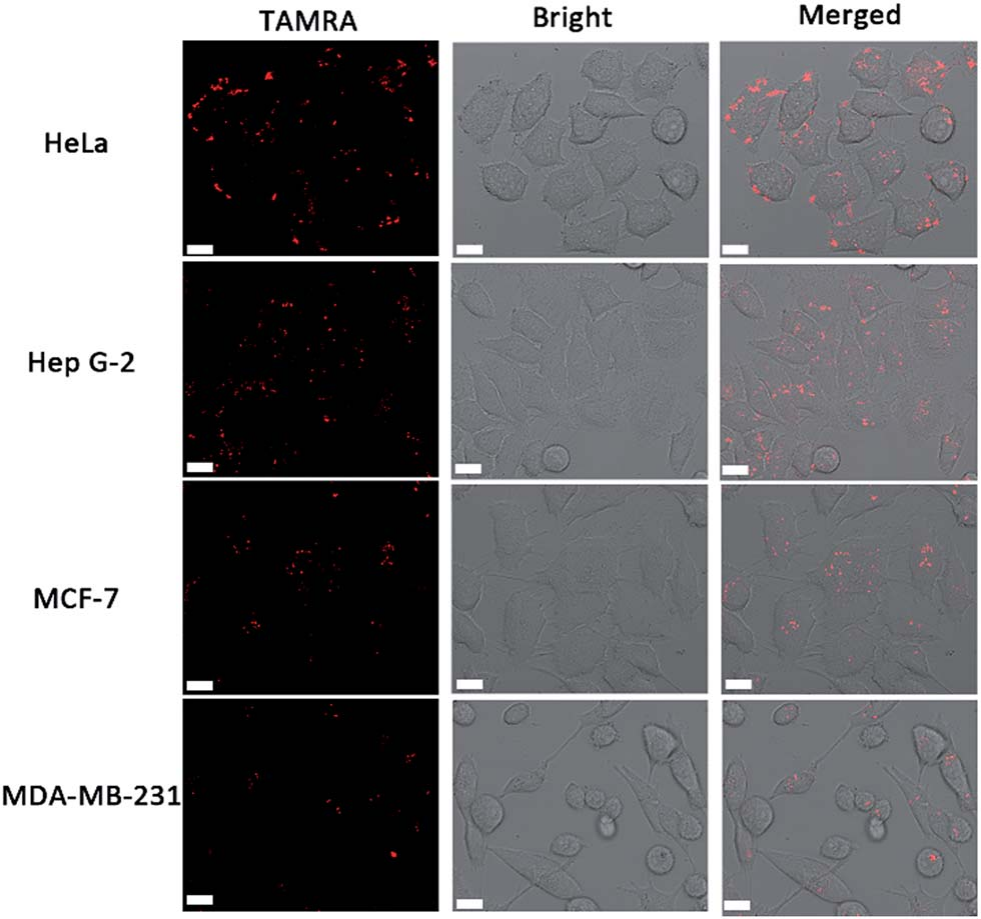
Confocal images of different cell lines using the cascade DNA logic gate to image intracellular telomerase activity. Scale bar: 15 μm.

In order to identify the telomerase activity of different cancer cells, a collection of 4 different cancer cell lines were used, including human cervical cancer cells (HeLa), human hepatocyte carcinoma cells (Hep G-2) and breast cancer cells (MCF-7 and MDA-MB-231). There is a general correlation between *hEST2* mRNA levels and telomerase activity.^33^ Different cancer cell lines show various *hEST2* mRNA levels, thus telomerase activities vary as well. As shown in Fig. 3, fluorescence signals can be observed in all of these cell lines, in accordance with the fact that elevated telomerase activity is observed in cancer cell lines. At the same time, the differences in observed fluorescence intensity (Fig. 3) show that the telomerase activity of the HeLa and HepG2 cells is higher than those of the two breast cancer cells (MCF-7 and MDA-MB-231), which is consistent with a previous report,^11^ suggesting the practicality of our proposed method in evaluating intracellular telomerase activity.

### Telomerase activity monitoring after EGCG treatment

**Fig. 4:**
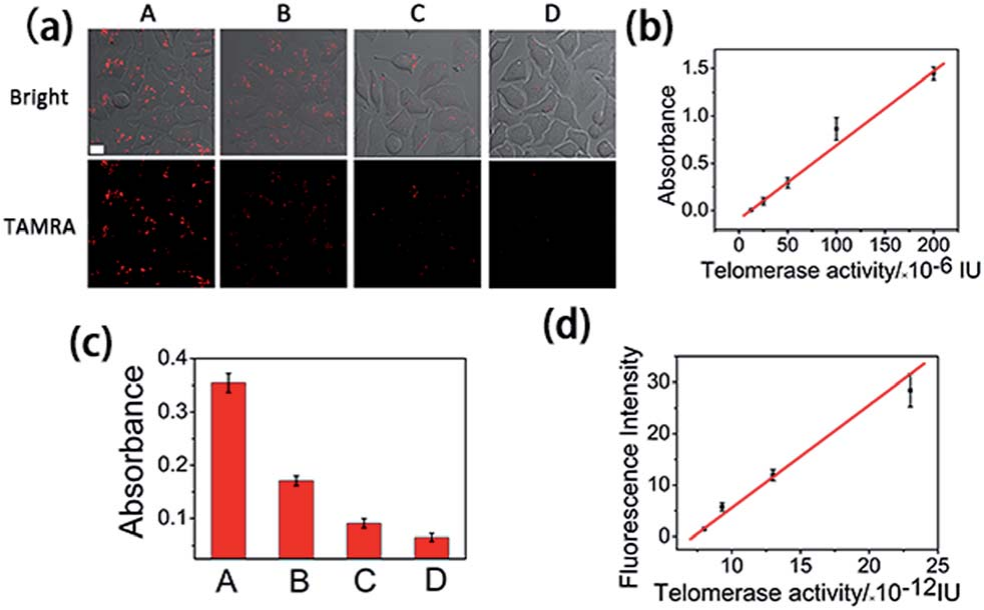
(a) Confocal images of HeLa cells treated with 0, 50, 100 or 200 μg mL^–1^ EGCG (from A to D) for 24 h and followed by transfecting with the logic gate for 3 h. (b) Standard curve of telomerase activity obtained using a commercial ELISA kit. (c) Absorbance of cell extracts detected using the ELISA kit after the HeLa cells were treated with 0, 50, 100 or 200 μg mL^–1^ EGCG (from A to D) for 24 h. (d) Calibration curve of the mean fluorescence intensity of a single cell, obtained from 20 cells of (a), *vs.* calculated mean telomerase activity of a single cell.

It has been reported that epigallocatechin-3-gallate (EGCG) could inhibit the telomerase activity of cancer cells.^34^ Thus, EGCG was used to treat HeLa cells to further test whether this method could be used to monitor the response of telomerase activity to EGCG treatment. HeLa cells were seeded in confocal dishes, and cultured with different concentrations of EGCG for 24 h. Confocal images (Fig. 4a) revealed a decreased fluorescence intensity with increased concentrations of EGCG from 0 to 200 μg mL^–1^. At the maximum tested concentration of 200 μg mL^–1^, the fluorescence signal totally disappeared, not only confirming the ability of EGCG to inhibit the telomerase activity of cancer cells, but also confirming the reliability of the proposed method in evaluating intracellular telomerase in response to pharmacological treatment.

### Intracellular telomerase activity quantification

Finally, we used HeLa cells to quantify intracellular telomerase activity. Cell extracts of different concentrations of EGCG treated HeLa cells were employed to correlate the fluorescence intensity in the confocal microscopy images (Fig. 4a) and the corresponding telomerase activity of the single cells measured using ELISA (Fig. 4b and c) using a calibration curve (Fig. 4d). With the increasing EGCG concentration, the telomerase activity in each single cell decreased, as did the fluorescence intensity. This has further been demonstrated using flow cytometry (Fig. S8†). The calibration curve displayed a linear relationship between fluorescence intensity and calculated intracellular telomerase activity of a single cell. The telomerase activity of a single HeLa cell was estimated to be 2.3 × 10^–11^ IU, which is in good agreement with previous reports,^23–25^ suggesting the reliability of the proposed method.

## Experimental

### Chemicals and materials

All the oligonucleotides used in this study (Table S1†) were synthesized and purified by Sangon Biotechnology Co. Ltd. (Shanghai, China), the modified sequences were HPLC purified, and other sequences without modification were ultraPAGE purified. A telomerase ELISA kit was obtained from Innovation Beyond Limits (Germany). Glycerol, deoxynucleotide triphosphates (dNTPs) solution mixture, 40% acrylamide mix solution, ammonium persulfate (APS), and 1,2-bis(dimethylamino)-ethane (TEMED) were obtained from Sangon Biotechnology Co. Ltd. (Shanghai, China). 4′,6-Diamidino-2-phenylindole (DAPI) was purchased from KeyGen Biotech. Co. Ltd. (Nanjing, China). 3-[(3-Cholamidopropyl)dimethylammonio]-1-propanesulfonic acid (CHAPS), Tween 20 and epigallocatechingallate (EGCG) were bought from Sigma-Aldrich (St. Louis, MO, USA). The transfection reagent, X-tremeGENE, was bought from Roche. All of the chemicals were of analytical grade and were used as received without any purification.

### Gel electrophoresis

10%, 7.5 cm non-denaturing polyacrylamide gels were used to purify the gate duplex. Electrophoresis was carried out at 120 V for 70 min at room temperature. After separation, the gel was stained using ethidium bromide and imaged with a fluorescence gel imaging system.

### Preparation and purification of gate duplex

The gates were prepared by mixing single oligonucleotide strands in 1× TAE/Mg^2+^ buffer (0.04 M Tris-acetate, 1 mM ethylenediaminetetraacetic acid (EDTA), and 12.5 mM magnesium acetate). The mixture was heated to 90 °C then slowly cooled down to room temperature over 1 h. For purification, a 10 μM mixture was loaded on the gel. After being separated using gel electrophoresis, the fully assembled duplex strands were excised and eluted for 24 h in 1× TAE/Mg^2+^ buffer. Gate concentration after purification was determined using UV-vis absorption spectroscopy as reported in ref. 35.

### Gate function examination using synthetic telomerase extension strands

Two input strands were added into the toehold-bearing duplex in TAE/Mg^2+^ buffer to obtain final concentrations of 800 nM and 200 nM for each. Three hours later, fluorescence was measured on the microplate reader in black 96-well plates (Corning). The TAMRA fluorophore was excited at 550 nm and the fluorescence was measured between 570 nm and 750 nm. The histogram was made by normalizing the positive control for each activated logic gate.

### Cell culture and telomerase extraction

HeLa cells, Hep G-2 cells, L-02 cells, MCF-7 cells and MDA-MB-231 cells were all obtained from KeyGen Biotech. Co. Ltd. (Nanjing, China). They were all seeded in DMEM medium (Gibco, Grand Island, NY) supplemented with 10% fetal calf serum (Gibco, Grand Island, NY), penicillin (100 μg mL^–1^), and streptomycin (100 μg mL^–1^) in 5% CO_2_, in a 37 °C incubator. Telomerase extracts were prepared according to the previous report with some modifications.^6^ HeLa cells were collected in the exponential phase of growth, and 10 million cells were dispensed in a 1.5 mL EP tube, washed twice with ice-cold phosphate buffered saline (pH 7.4) solution and resuspended in 200 μL of ice-cold CHAPS lysis buffer (10 mM Tris–HCl, pH 7.5, 1 mM MgCl_2_, 1 mM EGTA, 0.5% (W/V) CHAPS, 10% (V/V) glycerol). The solution was incubated on ice for 30 min and then centrifuged for 20 min (12 000 rpm, 4 °C). Without disturbing the pellet, the cleared lysate was carefully transferred to a fresh RNase-free tube, flash frozen and stored at –80 °C before use.

### Intracellular imaging of telomerase using the logic gate

Ten thousand cells were seeded in a 4-well glass bottom confocal dish and incubated for 12 h. Transfection was performed using 1 μL transfection reagent in 100 μL Opti-Mem at 37 °C for 3 h. The toehold-bearing DNA duplex and inputs were transfected at 50 nM and 200 nM respectively. Three hours later, the transfection mixture was removed from the confocal dishes and washed 3 times, then confocal microscopy images were obtained. For counter staining images, after replacing the transfection reagent, the nucleus was stained with DAPI using the standard protocol provided by the manufacturer. A 150 μL 300 nM DAPI (diluted with PBS) solution was added to the confocal dish and incubated for 5 min, then the sample was rinsed with PBS several times.

### Flow cytometry

HeLa cells were grown in a six-well culture plate for 24 hours. After thoroughly washing the cells with PBS, 1 mL Opti-Mem containing 10 μL transfection reagent, 50 nM toehold-bearing DNA duplex and 200 nM inputs was added to the cells and incubated for 3 hours. Then, the cells were washed with PBS and detached from the plate using trypsin. The cells were washed three times by adding PBS and centrifuging at 2000 rpm. Flow cytometry was performed using a Cytomics FC 500 MCL (Beckman coulter, USA) system under 488 nm excitation. Control cells incubated with only one input strand and the toehold-bearing DNA duplex were used for comparison. The measurement was controlled to stop either upon collecting 10 000 cells or reaching a measurement time of up to 5 min.

### EGCG treatment of HeLa cells

For confocal images, cells were seeded at 5000 per well in a 4-well glass bottom confocal dish and incubated with different concentrations of EGCG for 24 h, then transfected with the logic gate for 3 h, followed by confocal microscopy imaging. For detection using an ELISA kit, 10 million cells were incubated with different concentrations of EGCG for 24 h, and collected to extract telomerase. 50 μL of cell extract was subjected to the telomerase ELISA kit.

## Conclusions

In conclusion, we have developed a novel approach to image and quantify intracellular telomerase activity using DNA-based computation. In comparison with existing *in situ* detection methods, this assay does not require nanomaterial fabrication or modification processes. The practicality of the method to distinguish cancer cells from normal cells was demonstrated. Furthermore, the feasibility of the strategy has been tested in a range of different human cancer cell lines including cervical cancer cells, hepatocyte carcinoma cells, and breast cancer cells. In addition, the ability of this method to evaluate the response of intracellular telomerase activity to a telomerase-inhibiting model drug was also demonstrated. Consequently, the proposed method is a powerful and convenient analytical tool which has the potential to evaluate telomerase activity for clinical use and cancer diagnosis, and might help in screening telomerase-targeting drugs. The method also expands the application of DNA-based computation in cells.
