# Supplementary material for: Evaluation of intracellular telomerase activity through cascade DNA logic gates
Source: Chem Sci. 2016 Aug 1;8(1):174–80. doi: 10.1039/c6sc01953f (PMC5308402; doi:10.1039/c6sc01953f)
Supplement: Supplementary file 1 [file SC-008-C6SC01953F-s001.pdf]

Electronic Supplementary Material (ESI) for Chemical Science.
This journal is © The Royal Society of Chemistry 2016

Supporting Information

Supporting tables

Table S1. Oligonucleotide sequences used in telomerase based logic gate.

Name	Sequence (5'-3')
TS	AATCCGTCGAGCAGAGTT
G_F	TAMRA-CAATCTACAATCAAAGTGCTTCTGTTACTAAATCCGTCGAGCAGAGTT-P
G_T	AACCCTAACTCTGCTCGACGGATT
G_Q	TAGTAACAGAAGCACTTTGATTGTAGATTG-BHQ2
Input B	CGGATTTAGTAACAGAAGCACTTTGATTGTAGATTG
TS+1R	AATCCGTCGAGCAGAGTTAGGGTT
TS+2R	AATCCGTCGAGCAGAGTTAGGGTTAGGGTT
TS+3R	AATCCGTCGAGCAGAGTTAGGGTTAGGGTTAGGGTT

Underlined sequences were toehold regions. Letter 'P' in G_F indicated phosphate group modified. 'TAMRA' and 'BHQ2' indicated tetramethylrhodamine fluorophore and Black Hole Quencher modifications, respectively. 'TS+1R', 'TS+2R' and 'TS+3R' indicate that telomerase primer TS was extended by one, two and three repetitive nucleotide sequences (TTAGGG).

Supporting Figures

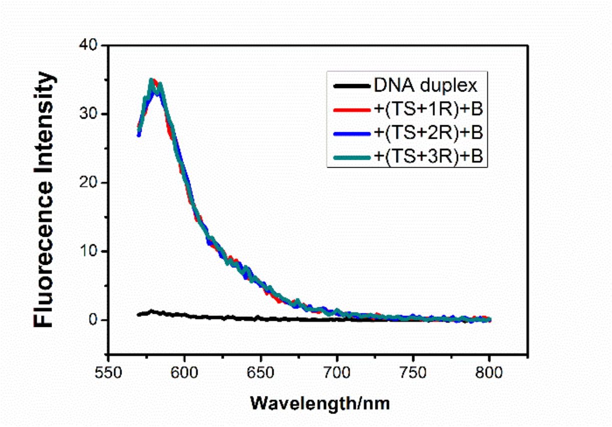

Fig. S1. Fluorescence spectroscopy recorded by microplate reader of the logic gate activation using synthetic TS oligonucleotide with different numbers of TTAGGG repeats and input B strands outside of cells. The excitation wavelength was 550nm.

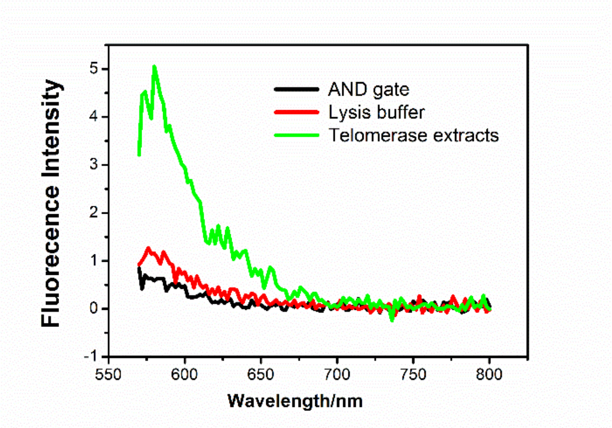

Fig. S2. Fluorescence spectroscopy recorded by microplate reader of AND logic gate including TS probe, input B and toehold-bearing duplex (black line), AND logic gate activation using lysis buffer only (red line) and telomerase from 5,000 cell extracts (green line). The excitation wavelength was 550nm.

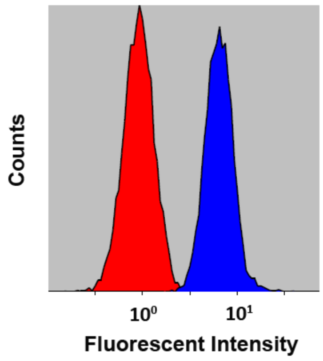

Fig. S3. Flow cytometry of HeLa cells transfected with logic gate for 3h. Red: Control sample of HeLa cells transfected with strand B and toehold-bearing DNA duplex; Blue: Positive sample of HeLa cells transfected with TS probe, strand B and toehold-bearing DNA duplex.

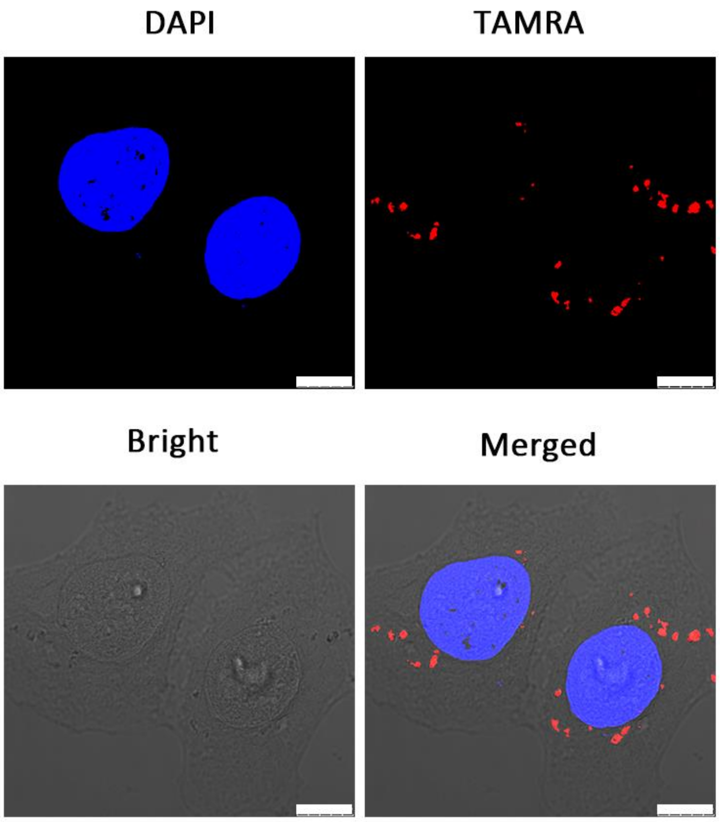

Fig. S4. Counter staining image of HeLa cells. Cells were transfected with TS probe, B strand and toehold-bearing DNA duplex for 3 h, then fixed and nucleus stained with DAPI, imaged by confocal microscopy. Scale bar: 10 μm.

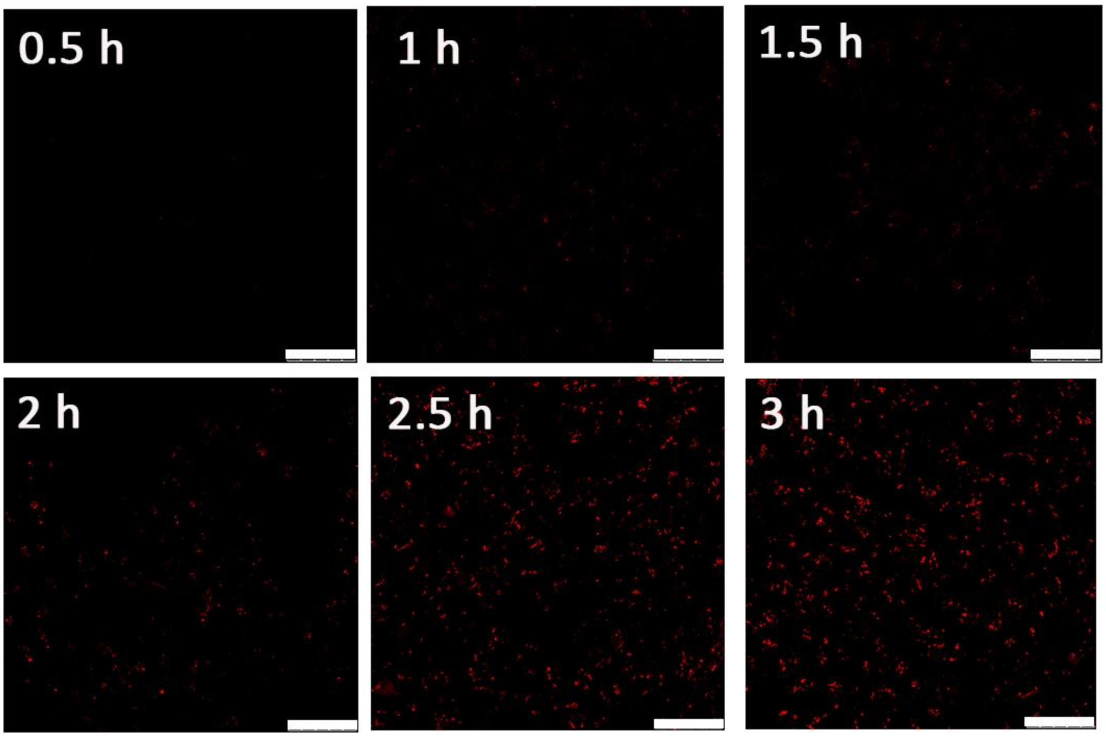

Fig. S5. Time course images of HeLa cells for intracellular telomerase activity detection by cascade DNA logic gate. HeLa cells were transfected with TS probe, strand B and toehold-bearing DNA duplex for different time and then imaged by confocal microscopy at specific time points. Scale bar: 75 μm.

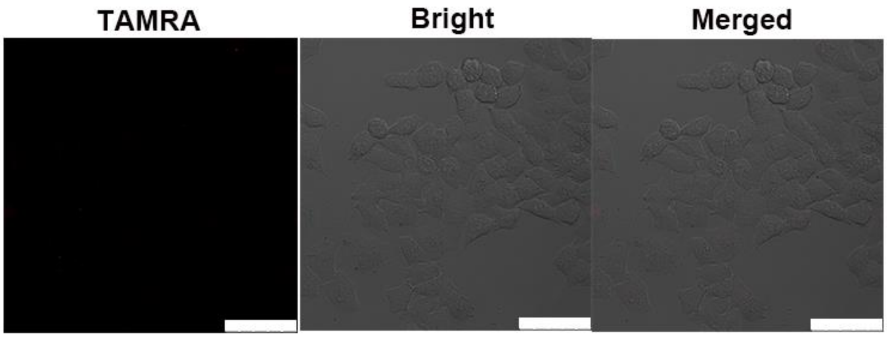

Fig. S6. Confocal image of HeLa cells transfected with toehold-bearing DNA duplex alone for 3 h. Scale bar: 75 μm.

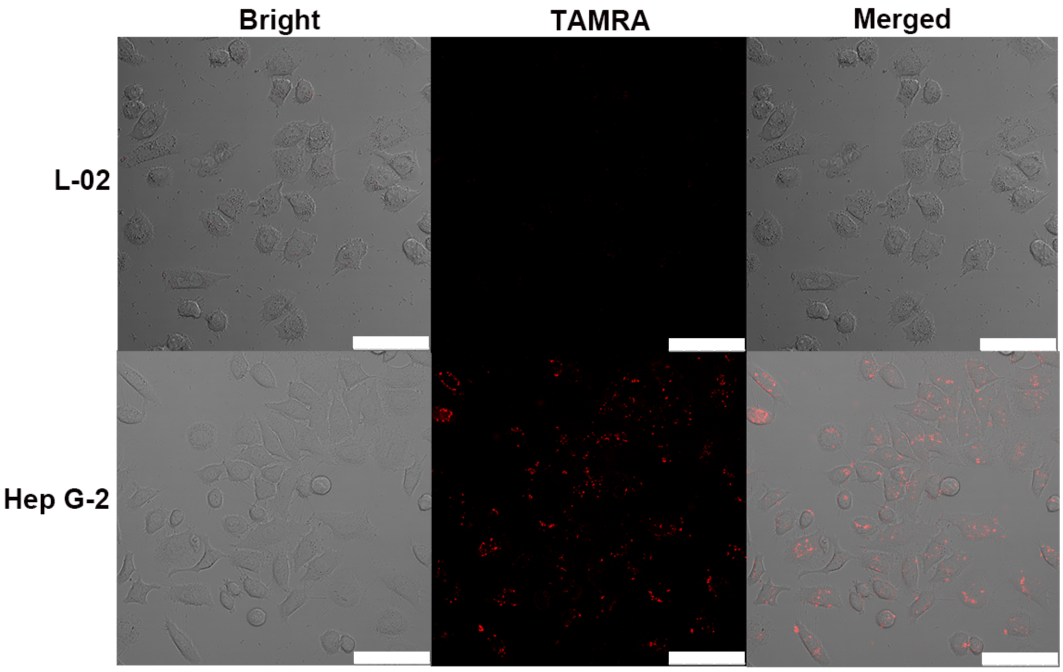

Fig. S7. Confocal image of L-02 and Hep G-2 cells transfected with DNA logic gate for 3 h. Scale bar was 75 μm.

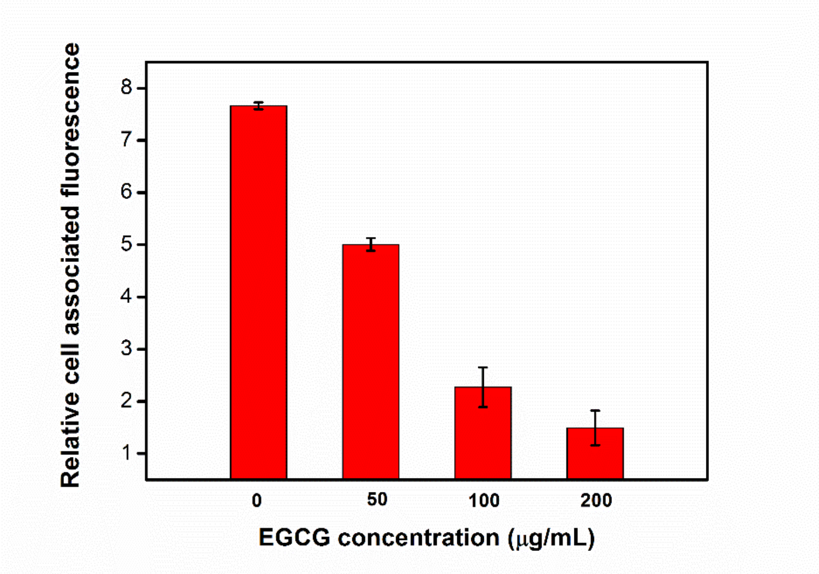

Fig. S8. Flow cytometry analysis of HeLa cells transfected with logic gate for 3h. The cells were treated with different concentration EGCG for 12 h before transfection. The fluorescence was normalized to the cell population transfected with toe-hold DNA duplex. Error bars represent the standard deviation from three independent measurements.
